# Comparison between two pedicle screw augmentation instrumentations in adult degenerative scoliosis with osteoporosis

**DOI:** 10.1186/1471-2474-12-286

**Published:** 2011-12-21

**Authors:** Yang Xie, Qiang Fu, Zi-qiang Chen, Zhi-cai Shi, Xiao-dong Zhu, Chuan-feng Wang, Ming Li

**Affiliations:** 1 Department of Orthopaedic Surgery, The Affiliated Changhai Hospital of The Second Military Medical University, 168 Changhai Road, Shanghai 200433, PR, China

**Keywords:** Degenerative scoliosis, Osteoporosis, Pedicle screw instrumentation augmentation, Polymethylmethacrylate, Autogenous bone

## Abstract

**Background:**

The operative treatment of adult degenerative scoliosis combined with osteoporosis increase following the epidemiological development. Studies have confirmed that screws in osteoporotic spines have significant lower-screw strength with more frequent screw movements within the vertebra than normal spines. Screws augmented with Polymethylmethacrylate (PMMA) or with autogenous bone can offer more powerful corrective force and significant advantages.

**Methods:**

A retrospective analysis was conducted on 31 consecutive patients with degenerative lumbar scoliosis combined with osteoporosis who had surgery from December 2000. All had a minimum of 2-year follow-up. All patients had posterior approach surgery. 14 of them were fixed with pedicle screw by augmentation with Polymethylmethacrylate (PMMA) and the other 17 patients with autogenous bone. Age, sex and whether smoking were similar between the two groups. Surgical time, blood loss, blood transfusion, medical cost, post surgery ICU time, hospital day, length of oral pain medicines taken, Pre-and postoperative Oswestry disability index questionnaire and surgical revision were documented and compared. Preoperative, postoperative and final follow up Cobb angle, sagittal lumbar curve, correction rate, and Follow up Cobb loss were also compared.

**Results:**

No significant differences were found between the autogenous bone group and Polymethylmethacrylate group with regards to all the targets above except for length of oral pain medicines taken and surgery cost. 2 patients were seen leakage during operation, but there is neither damage of nerve nor symptom after operation. No revision was needed.

**Conclusion:**

Both augmentation pedicle screw with Polymethylmethacrylate (PMMA) and autogenous bone treating degenerative lumbar scoliosis combined with osteoporosis can achieve a good surgical result. Less oral pain medicines taken are the potential benefits of Polymethylmethacrylate augmentation, but that is at the cost of more medical spending.

## Background

Degenerative scoliosis(DS) is one of the most prevalent findings in the elderly population. DS is defined as curvature of the spine in the coronal plane of greater than 10°Cobb angulation, which develops after skeletal maturity with a predominant lumbar location. DS is believed to develop as the result of osteoporosis, degenerative disc disease, osteoarthritic changes of the facet joints, compression fractures, and lateral olisthesis [1-6]. The patient often complains of low back pain and neurogenic claudication [7-9]. Surgery is indicated for severe back and/or radicular symptoms refractory to conservative treatment [7,10]. The operative treatment of DS is a formidable challenge to every spinal deformity surgeon. Narayan et al. [11] presented that in cases of posterolateral lumbar spinal fusion with pedicle screws, degenerative scoliosis has a lower (70%) fusion rate than degenerative disc disease alone (91%). In addition, senile osteoporosis makes pedicle screw instrumentation difficult to be anchored [9]. Pedicle screws rely primarily on cancellous bone for purchase, with the pedicle providing approximately 60% of the pullout strength [12]. Polymethylmethacrylate (PMMA) augmentation is regarded as an efficient way to enhance screw strength in osteoporotic bones [13,14].

Pedicle screw instrumentation by augmentation with PMMA is thought to be more effective than with autogenous bone in degenerative lumbar scoliosis combined with osteoporosis. However, using PMMA leads to relative more complications, such as bone cement leakage. Also this method may cause longer operation-time, more blood loss and more payment, etc. To our best knowledge, there is no study that compares these two techniques. The purpose of our study is to compare the results of pedicle screw by augmentation with PMMA versus with autogenous bone in degenerative lumbar scoliosis combined with osteoporosis. This is a retrospective, cohort study comparing the clinical and radiographic outcomes using two methods.

## Methods

**Figure 1:**
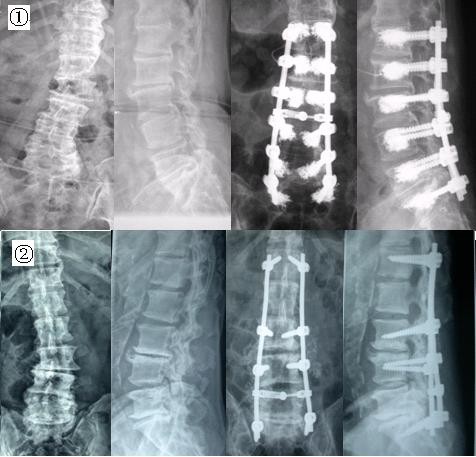
**Two cases of different screws augmented method**. Case 1: 61.6 years old, female, with 3 years back pain and radicular symptoms, post-approach decompression and spine fusion augmented with PMMA. Case 2: 68.1 years old, male, with 5 years back pain and radicular symptoms, post-approach decompression and spine fusion augmented with autogenous bone.

A total of 31 patients with degenerative lumbar scoliosis combined with osteoporosis underwent fusion with pedicle screw instrumentation from December 2000 to December 2006. All patients had Dual Energy X-ray examination for bone mineral density (BMD) in L1-L4 and were diagnosed as osteoporosis according to the WHO criteria (T < -2.5). The mean T-score was -3.03 ± 0.34 (-2.5 to -3.8). All the operations were practised by the same surgery group. There were 14 patients in PMMA group and 17 patients in autogenous bone group (see 2 cases in Figure 1). The average age of the patients was 60.9 ± 7.9 years (range 42.3-77.8). There were 6 men and 25 women. The average follow-up period was 3.8 ± 1.4 years (range 2.0-7.6 years). All had a minimum of 2-year follow-up.

Hospital records were reviewed for patients' medical comorbidities, smoking history, estimated intraoperative blood loss, operative time, and hospital stay. Hypertension, diabetes, heart disease, pulmonary disease, gastrointestinal disease, and kidney disease were considered to have medical comorbidities. The number of levels fused and the number of levels decompressed were measured. Complications were defined as any event for which the patient required specific treatment.

Anteroposterior and lateral radiographs were reviewed preoperatively, postoperatively and in final follow up periods. The Cobb angle and lumbar lordosis were assessed. Lumbar lordosis was measured from the upper endplate of L1 to the lower endplate of L5. Clinical outcomes were assessed with the Oswestry disability index. All patients were asked to fill Oswestry disability index scale perioperative and in the last follow-up.

The statistical analysis was performed using SPSS version 13.0. We used independent sample *t*-test. The significance was defined as *P *< 0.05.

### Operative procedures

Patients had undergone segmental pedicle screw instrumentation and autogenous iliac bone graft augmentation. 14 patients accepted the operation with PMMA augmentation.

For the autogenous bone graft augmentation group, using a burr, the pedicle entry point crossed by the longitudinal lateral edge of bony crest of superior articular process and the horizontal line that bisects the middle of the transverse process was prepared. A blunt tip 1.6 mm Kirschner-wire (K-wire) was gently tapped with mallet through the pedicle to the anterior cortex of vertebral body. The K-wire position was checked with fluoroscope image to make sure the K-wire was properly inside the pedicle and vertebral body, and then the K-wire was removed. A blunt tip 3-mm diameter K-wire was used to dilate the pedicle tract, and then it was removed.

The tract was palpated with a straight sensor probe to make sure the pedicle wall of the tract and the anterior cortex of the vertebral body were not violated. Then all the iliac bone graft were chipped to 2-3 mm and weighed by electronic balance. About 2 g graft was filled in to the pre-drilled pedicle screw tract for per screw. For the PMMA group, the preparation for the pedicel screw tract is the same. A 4 mm diameter bone biopsy needle (Allegiance, Healthcare Co.) was used for cement injection. About 2 ml PMMA was injected per screw with none requiring the use of autogenous iliac crest bone graft. Local bone graft (spinous processes) were used in all cases. Both of the two groups had decompression surgery at the level of spinal stenosis and had posterior or posterolateral fusion. All patients were braced with thoracolumbosacral orthosis for 3 months after surgery.

The autogenous bone group, the average number of levels fused was 6.0 ± 2.5 segments, ranging from 2 to 10 segments. The upper instrumented vertebra was T9 in 1 patient, T10 in 4 patients, T11 in 1 patients, L1 in 4 patients, L2 in 5 patients, and L3 in 2 patients,. The lower instrumented vertebra was L4 in 5 patient, L5 in 8 patients, and the sacrum in 4 patients.

The PMMA group, the average number of levels fused was 5.9 ± 1.9 segments, ranging from 3 to 9 segments. The upper instrumented vertebra was T9 in 1 patient, T10 in 2 patients, T11 in 1 patients, T12 in 1 patient, L1 in 5 patients, L2 in 3 patients, L3 in 1 patients. The lower instrumented vertebra was L4 in 3 patient, L5 in 8 patients, and the sacrum in 3 patients.

## Results

### Clinical evaluation

There were 14 patients in the PMMA group (13 female and 1 male), and the average age was 63.14(ranging 42.3-77.8). The average age in autogenous bone group(17 patient,12 female, 5 male) was 58.96 (range 50.4-72.8) and with no statistical difference (*P = *0.147) from the PMMA group (Table 1). Also there was no significant difference in smoking(*P = *0.194) and BMD(*P = *0.938) between the two groups.

**Table 1:** Demographics and baseline characteristics between Polymethylmethacrylate group and autogenous bone group

	Polymethylmethacrylate	Autogenous bone	*P*
Age(years)^1^	63.14 ± 8.98	58.96 ± 6.64	0.147

BMD(T value)^2^	2.98 ± 0.36	3.08 ± 0.34	0.938

Gender (n,%)^2^			

Female	13(92.9%)	12(70.6%)	0.103

Male	1(7.1%)	5(29.4%)	

Smoke (n,%)^2^			

Yes	11(78.6)	16(94.0%)	0.194

No	3(21.4%)	1(6.0%)	

^*P*-values are based on ^1^chi-square test and ^2 ^independent two samples *t *test

### Peri-operative evaluation

No significance were found between the groups in surgical time, blood loss, blood transfusion, post surgery ICU time and hospital day except for medical cost (157898.6 ± 46116.3 vs 121721.9 ± 45589.8, 0.037 < 0.05*) and length of oral pain medicines(5.5 ± 1.9 vs 13.1 ± 8.0, *P = *0.001 < 0.05*). (Table 2)

**Table 2:** Peri-operative Data

	Polymethylmethacrylate	Autogenous bone	*P*
Surgical time(min)	239 ± 45	246 ± 62	0.693

Blood loss(ml)	1828 ± 911	1758 ± 770	0.292

Blood transfusion (m1)	1421 ± 957	1188 ± 541	0.819

Post surgery ICU time(day)	1.0 ± 0.7	0.9 ± 0.5	0.427

Length for Hospital stay(day)	17.9 ± 10.2	19.6 ± 7.0	0.568

length of oral pain medicines taken(day)	5.5 ± 1.9	13.1 ± 8.0	0.001*

Length of fusion(n)	5.9 ± 1.9	6.0 ± 2.5	0.872

Treatment cost(RMB)	157898.6 ± 46116.3	121721.9 ± 45589.8	0.037*

*Indicates a significant difference. P < 0.05

### Radiological evaluation

No significance was found between the groups in preoperative, postoperative and final follow up Cobb angle, sagittal lumbar curve, correction rate. and Follow up Cobb loss. (Table 3)

**Table 3:** Radiographic Parameters

	Polymethylmethacrylate	Autogenous bone	*P*
Pre-operation Cobb angle(°)	23.8 ± 9.8	28.7 ± 10.6	0.200

Post-operation Cobb angle(°)	15.2 ± 6.2	17.9 ± 6.6	0.256

Cobb angle correction rate(%)	36.4 ± 13.5	35.7 ± 12.7	0.881

Follow up Cobb angle(°)	20.2 ± 7.2	17.0 ± 6.9	0.224

Follow up Cobb loss	1.8 ± 2.7	2.3 ± 2.7	0.605

Pre-operation sagittal lumbar curve(°)	34.4 ± 9.6	36.2 ± 12.5	0.659

Post -operation sagittal lumbar curve(°)	35.6 ± 3.9	34.6 ± 5.5	0.580

Follow up sagittal lumbar curve(°)	35.1 ± 5.2	37.6 ± 3.5	0.134

Follow up Cobb loss = Follow up Cobb angle - Post operation Cobb angle

### Clinical outcomes

The improvement of the Oswestry disability index was similar in both groups (*P = *0.313. The mean Oswestry disability index improved from 46.5 preoperatively to 18.7 at the last visit in the autogenous bone group with change rate of 57.9%, and in the PMMA group the mean Oswestry disability index improved from 49.8 preoperatively to 17.9 at the last visit with change rate of 62.7%.(Table 4)

**Table 4:** Clinical outcomes

	Polymethylmethacrylate	Autogenous bone	*P*
Pre-operation	49.8 ± 11.5	46.5 ± 13.0	0.465

Post-operation	17.9 ± 6.2	18.7 ± 5.3	0.715

ODI change Rate(%)	62.7 ± 13.2	57.9 ± 12.6	0.313

The numbers of medical co-morbidities were similar in both groups. Medical co-morbidities included hypertension in 10 patients, heart disease in 5 patients, DM in 4 patients, arthritis deformans disease in 2 patients.

Complications were shown in Table 5. The numbers of complications were similar in both groups. There were 1 pneumonia, 1 urinary tract infection and 1 wound infection. All 3 patients were cured through antibiotics treatment. 2 patients were seen leakage during operation, but it didn't cause any damage of nerve or symptom after operation. No revision was needed.

**Table 5:** Complication

Complication	Polymethylmethacrylate	autogenous bone
pneumonia	1	0

Neurologic defict	0	0

Urinary tract infection	0	1

Wound infection	0	1

Leakage	2	--

Pseudarthrosis	0	0

Loosening of screws	0	0

Breakage of screws	0	0

Pain of iliac bone	--	4

Revision	0	0

## Discussion

Pedicle screws are the workhorse of spinal instrumentation for the adult spine. Not long ago, osteoporotic patients with progressive deformity or fracture, even with neurologic manifestations, were considered inoperable by many spine surgeons for the reason that fixation strength of the pedicle screws decreases in frail spines of elderly osteoporotic patients [15-17]. With advances in surgical and instrumentation techniques, various methods have been used to improve the fixation strength. Pedicle screws rely primarily on cancellous bone for purchase, with the pedicle providing approximately 60% of the pullout strength [12].

Fixation in marginal bone was more regularly successful through augmentation of screw fixation with autogenous bone or bone cement [18]. The augmentation of pedicle screw fixation with various bone cements such as PMMA, calcium phosphate and calcium sulfate cement has been studied to address potential solutions to inadequate fixation [19-21]. Polymethylmethacrylate is the most commonly used bone cement in spinal procedures today [13,14,22]. Some studies demonstrated that PMMA has stronger augmentation power than calcium phosphate and calcium sulfate cement in primary screw augmentation [23,24]. In most of the experimental studies, the amount of various cement injection was 1 to 3 ml, and the pull-out strength of pedicle screw can be significantly increased up to 147% to 300% [23-26]. Chang et al. [27] did a retrospective study to evaluate the clinical results of patients with osteoporosis and various spinal diseases treated surgically with PMMA augmented pedicle screw. In their study there was neither neurologic deterioration nor symptomatic cement leakage after surgery. Evans et al. [28]conducted a study to investigate the effect of using either a low-viscosity bone cement(Palacos LV) or a bone augmentation material(Cortoss) on the pullout strength of pedicle screws. The pullout strength of the non-augmented screws was 1203 ± 260 N, while the pullout strength of the augmented screws was 1970 ± 220 N(Palacos LV) and 2021 ± 342 N(Cortoss). Both Palacos LV and Cortoss significantly increased the pullout strength. There was no significant difference between the Palacos LV and Cortoss groups. Pain was significantly released and kyphotic deformity was significantly corrected. The average loss of kyphosis correction was 3° at the final follow up. There was no significant screw migration when the screws distances just after operation and at the final follow-up were compared. So they considered the technique of PMMA for augmentation of pedicle screw is a safe, reliable, and practical technique for osteoporotic patients who also had various spinal diseases and need spinal instrumentation. Burval et al. take a research to compare the pullout strengths of pedicle screws fixed in osteoporotic vertebrae using polymethyl methacrylate delivered by 2 augmentation techniques, a standard transpedicular approach and kyphoplasty type approach. They found that pedicle screw augmentation with polymethyl methacrylate improves the initial fixation strength and fatigue strength of instrumentation in osteoporotic vertebrae. Pedicle screws augmented using the kyphoplasty technique had significantly greater pullout strength than those augmented with transpedicular augmentation technique and those placed in healthy control vertebrae with no augmentation [17]. However, the use of bone cement is associated with difficulty in removing the screw and bone cement leakage into the spinal canal or neural foramina.

In our study, we compared these two techniques of autogenous bone or bone cement augmentation of screw fixation. Pedicle screw instrumentation by augmentation with PMMA didn't show more effective than with autogenous bone in degenerative lumbar scoliosis combined with osteoporosis as we ever thought. Both can achieve a similar curve corrective force. Also the operation result is similar according to ODI scores. However, using PMMA leads to relative more complications, such as cement leakage, but all of them were spotty or linear minor leakages without any sign of neurovascular injury. Although the direct injection method with cement amount of 2 to 3 ml for each pedicle is safe, fluoroscope monitoring during the cement injection is suggested to avoid major cement leakage.

The two methods had similar operation-time and blood loss. The reasons for this result may be that although the injection of cement may cause more time cost and blood loss but the procedure for taking iliac bone even prolong the operation time and cause more blood loss. The intraoperative blood loss was 1828 ± 911 in PMMA group and 1758 ± 770 in autogenous bone group. The reason for such large amount of blood loss may be as follows: a) All the patients received a long post-exposure and augmentation either with PMMA or autogenous bone. The augmentation was a little bit time consuming for us due to the learning curve for this technique. The surgical time is certainly longer than surgery without augmentation and may caused more blood loss. b) The process to get autogenous bone graft from supply area of iliac bone also increace blood loss. C)The fusion levels for some of the patients was as long as 10 segments suggested a really long fusion.

In this study, shorter time of oral pain medicines taken was seen in Polymethylmethacrylate augmentation group. The main pain caused by atuogenous bone group comes from the supply area of iliac bone, and this kind of pain caused as long as 3-4 weeks drug apply. Also, this difference could be attributed to the treatment of the pain caused by osteoporosis through the use of Polymethylemethacrylate. Although more analgetic is needed, the whole medical cost for autogenous bone augmentation is less than PMMA augmentation. The additional high price of PMMA may be the main reason.

## Limitations

a) In this group of patients with osteoporosis, we all use augmentation either with PMMA or autogenous bone and we didn't set a control group which didn't use any anugmentation. So it was not clear of the difference between patients with or without augmentation. b) BMD using DXA in the lumbar spine is a weak methodological point as the BMD measured in spine may be affected by the degenerative and sclerotic changes created by the deformity, vertebral rotation and compression of the vertebra. Biomechanical adaptation within the cancellous or cortical bone tissue in adult scoliosis patients may influence the value[29,30]. c) Patients in the present research was heterogeneous such as short and long instrumentation were chosen individually.d) The present study may be limited by the small sample size and the strict criteria for patient selection.

## Conclusions

Degenerative lumbar scoliosis combined with osteoporosis treated with pedicle screw by augmentation with Polymethylmethacrylate (PMMA) or autogenous bone both can achieve a good surgical result. Both of these two technique are safe, reliable, and practical. Less oral pain medicines taken are the potential benefits of Polymethylmethacrylate augmentation, but that is at the cost of more medical spending.

## Competing interests

The authors declare that they have no competing interests.

## Authors' contributions

Yang Xie, Qiang Fu and Zi-qiang Chen contributed to the study concepts, study design, literature research and manuscript preparation. Ming Li is the guarantor of integrity of the entire study and participated in the operation and manuscript review. Chuan-feng Wang participated in literature research and clinical studies. Zhi-cai Shi and Xiao-dong Zhu participated in data acquisition and data analysis. All authors read and approved the final manuscript.

## Pre-publication history

The pre-publication history for this paper can be accessed here:

http://www.biomedcentral.com/1471-2474/12/286/prepub
